# A novel nomogram based on inflammation biomarkers for predicting radiation cystitis in patients with local advanced cervical cancer

**DOI:** 10.1002/cam4.7245

**Published:** 2024-05-24

**Authors:** Jie Lin, Jiexiang Lin, Linying Liu, Ning Xie, Haijuan Yu, Sufang Deng, Yang Sun

**Affiliations:** ^1^ Department of Gynecology Clinical Oncology School of Fujian Medical University, Fujian Cancer Hospital Fuzhou Fujian China; ^2^ Shengli Clinical Medical College of Fujian Medical University Fuzhou Fujian China; ^3^ Department of Urology Fujian Provincial Hospital Fuzhou Fujian China

**Keywords:** cervical cancer, nomogram, PAR, radiation cystitis, radiotherapy, SII

## Abstract

**Backgrounds:**

Platelet‐to‐albumin ratio (PAR) is a new systemic inflammatory prognostic indicator associated with many inflammatory diseases. However, its role in radiation cystitis (RC) is obscure. This study aimed to explore whether PAR could be used as an effective parameter for predicting the RC risk in local advanced cervical cancer (CC) treated with radiotherapy.

**Methods:**

A total of 319 local advanced CC patients who received radical radiotherapy at Fujian Cancer Hospital were enrolled between December 2018 and January 2021. Demographics and clinical parameters were retrospectively analyzed. Univariate and multivariate analyses were used to identify the risk factors for RC. Backward and stepwise regression was applied to construct two monograms‐one with primary significant factors and the other with extra inflammatory biomarkers. A DeLong test was applied to compare the prediction abilities of two nomograms. Calibration curves and decision curve analysis (DCA) evaluated its prediction consistency, discrimination ability, and clinical net benefit.

**Results:**

Univariate analysis showed that age, tumor size, stage, total radiation dose, pelvic radiation dose, Systemic Immune‐Inflammation Index (SII), platelet‐to‐lymphocyte ratio (PLR), and PAR were significantly associated with RC occurrence (all *p* < 0.05). Multivariate analyses indicated that age, tumor size, stage, total radiation dose, and PAR were independent factors (all *p* < 0.05). Then, the area under curve (AUC) value of the nomogram_SII+PAR_ was higher (AUC = 0.774) compared to that of the baseline nomogram (AUC = 0.726) (*p*_Delong_ = 0.02). Also, the five‐cross validation confirmed the stability of the nomogram_SII+PAR_. Moreover, the calibration curve and DCA exhibited the nomograms' good prediction consistency and clinical practicability.

**Conclusions:**

PAR and SII could be valued for CC patients who are treated with radiation therapy. The nomogram based on PAR and SII could stratify patients who need extra intervention and nursing care to prevent bladder radiation damage and improve patients' quality of life.

## 1 INTRODUCTION

Cervical cancer (CC) is a global public health problem, with an exceptionally high burden in many low‐and middle‐income countries.^1^ More than half a million women are diagnosed with CC every year, resulting in over 300,000 deaths worldwide.^2^ Except for early disease treated with surgery, external beam radiotherapy combined with brachytherapy are primary treatments for CC patients.^3^ Brachytherapy could be a preoperative option for early‐stage tumors with local risk factors. Postoperative radiotherapy is indicated according to histopathological criteria. Concurrent chemoradiotherapy (CCRT) is the standard treatment for advanced local tumors.^4^

Radiotherapy uses ionizing radiation to target and kill tumor tissue, but normal surrounding tissue can also be damaged.^5^ Since the bladder is located next to the cervix, it inevitably absorbs the poisonous spectrum during radiation. Despite technical advances in radiation oncology to minimize radiation scatter to normal tissue, such as high‐energy linear accelerators, conformal radiation therapy, and intensity‐modulated radiation therapy, RC incidence still ranges from 5% to 50% and remains stable over time.^6^, ^7^ Moreover, the therapeutic possibilities are limited. Hence, it is crucial to build a warning system for predicting RC occurrence before pelvic radiation and to take interventions in advance for RC prevention.

Radiation cystitis (RC) results from inflammation, fibrosis, and vascular damage of the bladder following pelvic radiotherapy, which could be defined by the Radiation Therapy Oncology Group (RTOG) classification.^8^ Different degrees of low urinary tract symptoms and hematuria are the main clinical presentation. RC was divided into acute RC, typically occurring within 3–6 months of radiation, and late RC after 6 months to 20 years after radiation therapy.^9^ Acute RC is usually self‐limiting and is generally managed conservatively. Late RC, especially hematuria, on the other hand, is challenging to deal with and seriously impacts patients' life quality and, in very severe cases, becomes life‐threatening. Due to the limitations in therapeutic strategies, the importance of the meticulous selection of RC patients before radiation should be highlighted to prevent its occurrence.

Recently, several integrated indexes have been used for the assessment of inflammatory diseases and various cancers, such as platelet‐to‐albumin ratio (PAR), systemic immune‐inflammation index (SII), neutrophil‐to‐lymphocyte ratio (NLR), and platelet‐to‐lymphocyte ratio (PLR).^10^ Transferring these markers into exercise physiology seems highly beneficial and efficient, especially due to the time‐saving and economic benefits of assessment and calculation.^11^ Herein, we aimed to investigate the effectiveness of systemic inflammatory markers in risk‐stratifying RC patients.

## 2 MATERIALS AND METHODS

### 2.1 Patients

**FIGURE 1:**
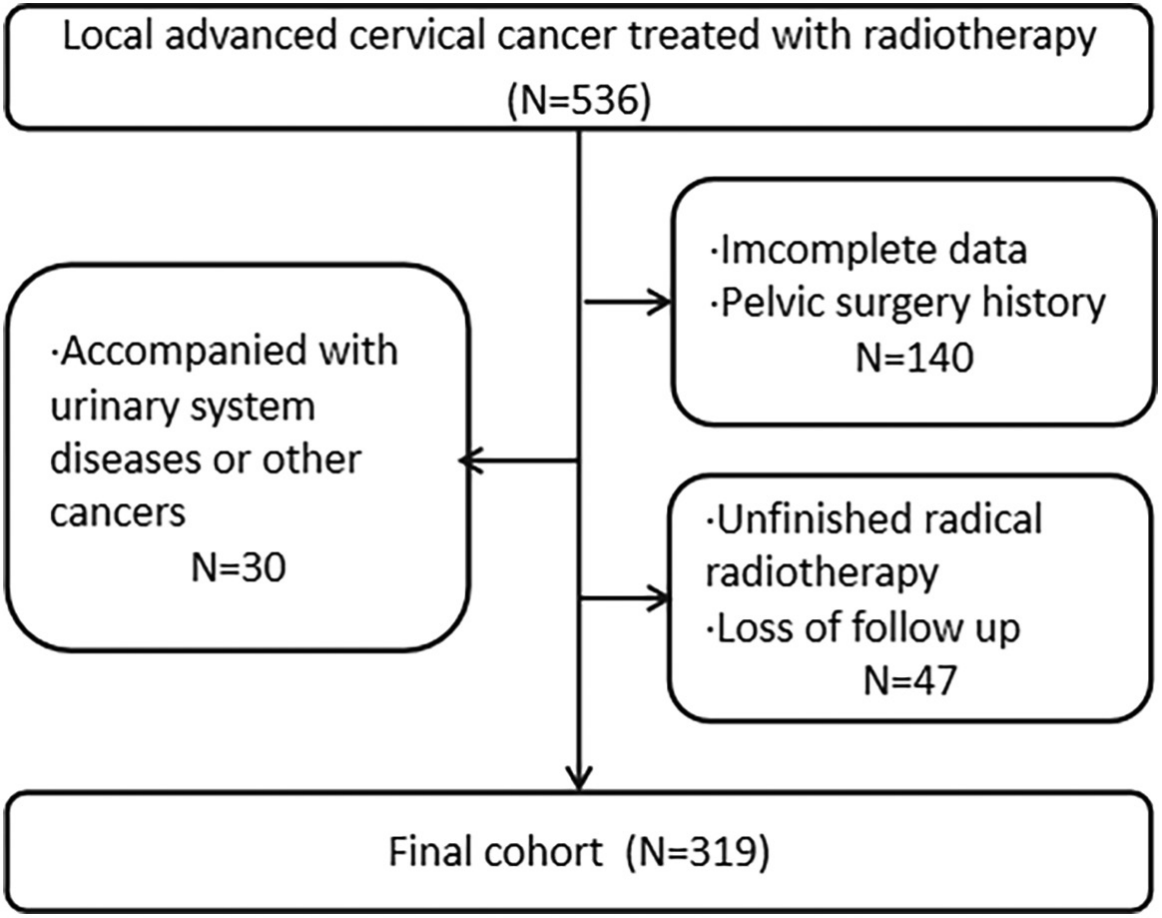
Patient selection.

Five hundred thirty‐six eligible CC patients with stage IB‐IVa (2018 International Federation of Gynecology and Obstetrics (FIGO) Staging Classification) who received radiation therapy in Fujian Cancer Hospital between December 2018 and January 2021 were retrospectively enrolled. The inclusion criteria were: (1) CC was confirmed by pathological biopsy; (2) patients underwent radical radiation with or without chemotherapy; and (3) the patient's age ranges from 18 to 80. We excluded (1) patients with incomplete or duplicate clinical data; (2) patients with pelvic surgery history; (3) patients with a history of urinary system diseases or other malignancies; (4) patients with unfinished radical radiotherapy; and (5) patients without complete follow‐up information. Finally, 319 patients were included. Figure 1 demonstrates the detailed screening process.

### 2.2 Data collection

Patient medical records were retrospectively reviewed, including age, BMI, diabetes, hypertension, tumor size, grade, MRI data, and routine blood tests, such as lymphocytes, platelets, and albumin, on admission day. Tumor sizes were calculated by two experienced radiologists using MRI images. We calculated the PAR, SII, PLR, NLR, and PNI based on the formula as follows: PAR = platelet counts (10^9^/L) divided by albumin levels (g/L); SII = (neutrophil count × platelet count)/lymphocyte count; PLR = platelet count/lymphocyte count; NLR = neutrophil count/lymphocyte count.^10^, ^12^ PNI = albumin level (g/L) + 5 × total lymphocyte count (10^9^/L).^13^

### 2.3 Radical radiotherapy

Patients received external pelvic beam radiotherapy (EBRT) combined with individualized high‐dose rate intracavitary brachytherapy (HDR‐ICBT). The EBRT was conducted 25–28 fractions of 1.8–2.0 Gy each time to get a total dose of 45.0–50.0 Gy. An extra dose (10–16 Gy) was added for positive pelvic or para‐aortic lymph nodes. Then, HDR‐ICBT was conducted to get a total of 28 Gy (Dose: 7.0 Gy per time; Time interval: 7 days for 4 weeks). Total RT dose (EQD2, Equivalent Dose in 2 Gy fractions) was calculated based on EBRT (EQD2) dose and HDR‐ICBT (EQD2). EQD2 = D* (*d* + *α*/*β*)/(2 + *α*/*β*). *D* refers to the actual dose, *d* is the dose for each fraction, and *α*/*β* is a correction factor.^14^

### 2.4 Chemotherapy

Based on NCCN (National Comprehensive Cancer Network) guidelines,^3^ cisplatin‐based CCRT was recommended unless infeasible. The chemotherapy regimens included cisplatin (40 mg/m^2^) or nedaplatin (80 mg/m^2^) monotherapy or combined with paclitaxel (135 mg/m^2^) every 3 weeks during RT. Some patients only received RT for old age, physical conditions, fear, economic status, or other reasons.

### 2.5 Follow‐up

Patients were recommended for follow‐up visits once every 3 months in the first 2 years, every 6 months for 3–5 years, and then once annually. Telephone calls were also used for data collection. The last follow‐up time is June 2023.

### 2.6 Criteria of radiation cystitis

RC describes the inflammation and cellular destruction of the bladder as an adverse effect of pelvic radiation without other explainable causes like genitourinary tumors, infection, or drug‐induced cystitis.^15^ Milder symptoms include increased frequency, urgency, and dysuria. Severe symptoms include urinary incontinence, gross hematuria, and damage progression, including fistula formation and necrotic bladder tissue.^16^ Based on the toxicity criteria of the European Organisation for Research and Treatment of Cancer/Radiation Therapy Oncology Group (EORTC/RTOG), patients who were rated ≥Grade 1 were included.^8^ Patients with histories of urinary system disease or urinary bacteria were excluded.

### 2.7 Statistical analysis

R (Version 4.2.1) (https://www.r‐project.org) was used for statistical analysis. The chi‐square test was used to compare the clinicopathological categorical variables. Univariate and multivariate Logistic regression analysis was employed to estimate the risk factors related to RC. Risk factors in univariate analysis (*p* < 0.05) were put into the backward stepwise selection to construct two nomograms—one with primary significant factors and the other with extra inflammatory biomarkers. AUC was used to assess the performance. A DeLong test^17^ was applied to compare the AUC values of two nomograms to see whether inflammatory biomarkers could improve the predictive ability. The stability and credibility of the nomogram with inflammatory biomarkers were assessed by fivefold validation. Calibration plots used 200 bootstrap resamples to decrease the overfitting bias. Decision curve analysis (DCA) was calculated by the net benefit over a spectrum of probability thresholds. All statistical tests were two‐sided. *p* < 0.05 was statistically significant.

## 3 RESULTS

### 3.1 Baseline characteristics

The demographic and clinical characteristics of the studied patients (RC and non‐RC groups) are described in Table 1. The median follow‐up time was 41.9 months (32.5, 47.7). The mean age was 57 (55, 66) years old. The average tumor size was 4.3 cm (3.5, 5.3). Among these patients, 39.18% were in stage II, and 58.62% were in stage III. 79.94% of patients received concurrent radiotherapy. In contrast, 20.06% were treated by radiotherapy alone. Neoadjuvant chemotherapy was only applied in a tiny proportion (4.39%).

**TABLE 1:** Demographic details and baseline.

	Total (*n* = 319)	Non‐RC (*n* = 267)	RC (*n* = 52)
Age, years (IQR)	57 (52, 66)	57 (52, 65)	61 (40, 70)
BMI (IQR)	22.64 (20.57, 24.75)	22.64 (20.60, 24.99)	22.66 (20.52, 24.41)
Hypertension	72 (22.57)	59 (22.10)	13 (25.00)
Diabetes	28 (8.78)	23 (8.61)	5 (9.62)
Tumor size, cm (IQR)	4.3 (3.5, 5.3)	4.3 (3.5, 5.3)	4.6 (3.5, 5.4)
Tumor stage *n* (%)			
I	3 (0.94)	3 (1.12)	0 (0)
II	125 (39.18)	111 (41.57)	14 (26.92)
III	187 (58.62)	151 (56.55)	36 (69.23)
IV	4 (1.25)	2 (0.75)	2 (3.85)
Radiotherapy plan *n* (%)			
RT	64 (20.06)	50 (18.73)	14 (26.92)
CCRT	255 (79.94)	217 (81.27)	38 (73.08)
NACT, *n* (%)			
No	305 (95.61)	255 (95.51)	50 (96.15)
Yes	14 (4.39)	12 (4.49)	2 (3.85)

Abbreviations: CCRT, concurrent chemoradiotherapy; IQR, Interquartile range; NACT, neoadjuvant chemotherapy; RC, radiation cystitis; RT, radiotherapy.

### 3.2 Independent risk factors for radiation cystitis

**FIGURE 2:**
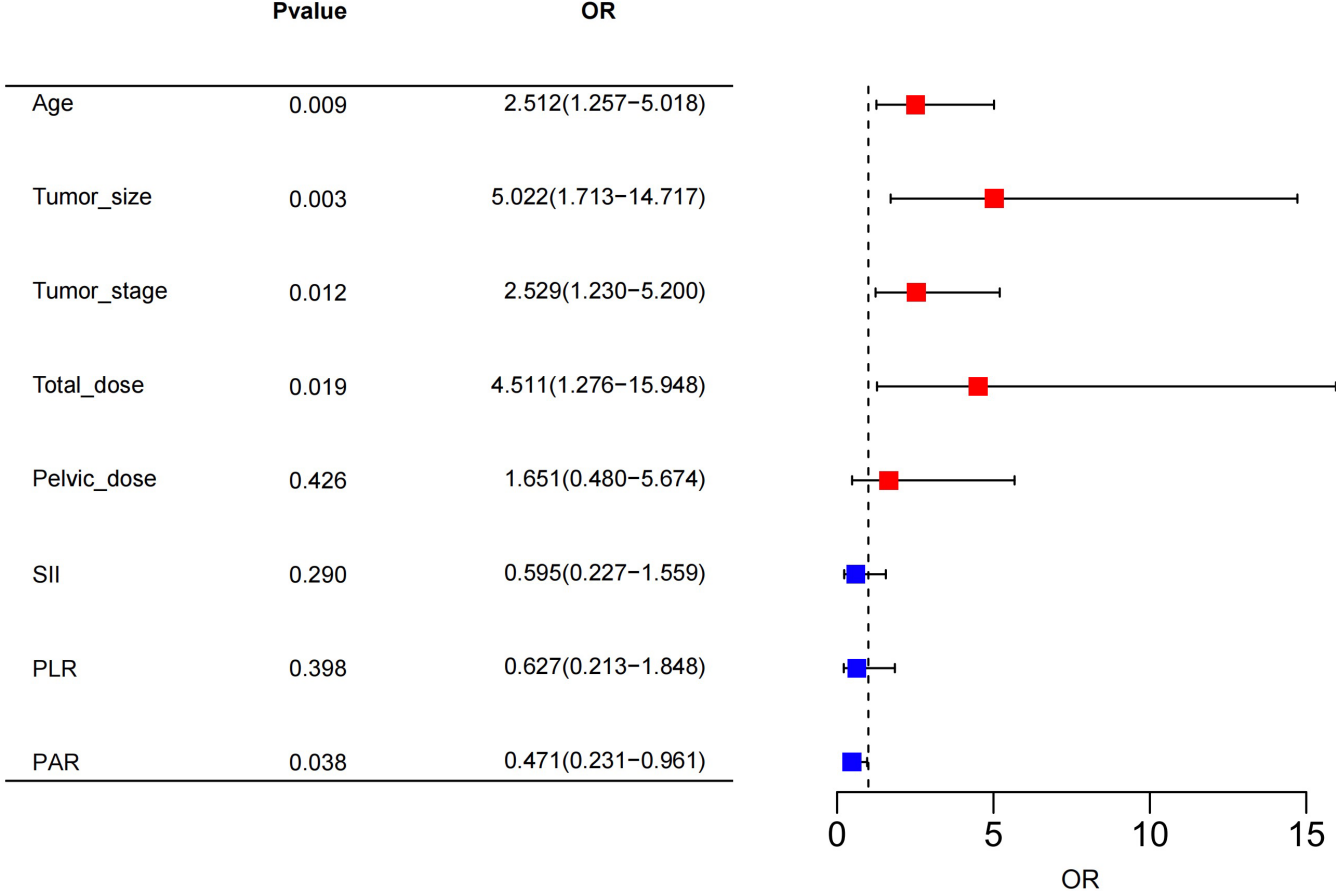
Multivariate logistic regression analysis of the nomogram. Blue square: OR < 1; Red square: OR > 1.

Univariate analysis showed that age (*p* = 0.016), tumor size (*p* = 0.039), stage (*p* = 0.034), total radiation dose (*p* = 0.005), pelvic radiation dose (*p* = 0.017), SII (*p* = 0.039), PLR (*p* = 0.012) and PAR (*p* = 0.015) were significantly associated with the RC occurrence. However, only age (*p* = 0.009, odds ratio [OR] = 2.512 [1.257–5.018]), tumor size (*p* = 0.003, OR = 5.022 [1.713–14.717]), stage (*p* = 0.012, OR = 2.529[1.230–5.200]), total radiation dose (*p* = 0.019, OR = 4.511 [1.276–15.948]), and PAR (*p* = 0.038, OR = 0.471 [0.231–0.961]) were shown to be independent prognostic factors by multivariate analysis (Table 2; Figure 2).

**TABLE 2:** Univariate and multivariate logistic regression analysis.

	Construction cohort, *n* = 319		Univariate analysis		Multivariate analysis	
	Non‐radiation cystitis, *n* = 267	Radiation cystitis, *n* = 52	OR	*p*	OR	*p*
Age, years						
<65	198	30	2.104 (1.138–3.891)	0.016*	2.512 (1.257–5.018)	0.009**
≥65	69	22				
BMI, (IQR)						
<22.13	115	20	1.211 (0.658–2.226)	0.538	–	–
≥22.13	152	32				
Hypertension						
N	208	39	1.175 (0.589–2.345)	0.647	–	–
Y	59	13				
Diabetes						
N	244	47	1.129 (0.408–3.118)	1.000	–	–
Y	23	5				
Tumor size, cm						
<6.75	251	44	2.852 (1.151–7.066)	0.039*	5.022 (1.713–14.717)	0.003**
≥6.75	16	8				
Tumor stage						
I，II	114	14	2.022 (1.046–3.909)	0.034*	2.529 (1.230–5.200)	0.012*
III，IV	153	38				
Radiotherapy plan						
RT	50	14	0.625 (0.315–1.241)	0.177	–	–
CCRT	217	38				
Total radiation dose, Gy						
≤87	68	4	4.101 (1.426–11.794)	0.005*	4.511 (1.276–15.948)	0.019*
>87	199	48				
Single radiation dose, Gy						
≤180	143	24	1.345 (0.741–2.442)	0.328	–	–
>180	124	28				
ERBT fraction						
≥25	252	50	0.672 (0.149–3.031)	0.855	–	–
<25	15	2				
ERBT dose, Gy						
≤46.34	59	4	3.404 (1.179–9.826)	0.017*	1.651 (0.480–5.674)	0.426
>46.34	208	48				
HDR‐ICBT dose, Gy						
≤39.69	103	14	1.705 (0.881–3.300)	0.111	–	–
>39.69	164	38				
Neoadjuvant chemotherapy						
N	255	50	0.850 (0.185–3.915)	1.000	–	–
Y	12	2				
WBC, 10^9^						
<9	224	39	1.736 (0.856–3.523)	0.123	–	–
≥9	43	13				
Hb, g/L						
≤126	135	31	0.693 (0.379–1.267)	0.232	–	–
>126	132	21				
PLT, 10^9^						
<360	216	47	0.451 (0.171–1.190)	0.100	–	–
≥360	51	5				
Neutrophils, 10^9^						
<4.2	141	24	1.306 (0.720–2.369)	0.380	–	–
≥4.2	126	28				
Lymphocyte, 10^9^						
≤1.8	95	19	0.959 (0.517–1.779)	0.895	–	–
>1.8	172	33				
Alb, g/L						
≤44.1	219	38	1.681 (0.845–3.344)	0.136	–	–
>44.1	48	14				
SII						
≤757	171	41	0.478 (0.235–0.973)	0.039*	0.595 (0.227–1.559)	0.290
>757	96	11				
PNI						
≤57.1	234	43	1.484 (0.663–3.322)	0.459	–	–
>57.1	33	9				
NLR						
≤2.23	210	39	1.228 (0.614–2.455)	0.560	–	–
>2.23	57	13				
PLR						
≤181	190	45	0.384 (0.166–0.888)	0.021*	0.627 (0.213–1.848)	0.398
>181	77	7				
PAR						
≤6.03	91	27	0.479 (0.263–0.872)	0.015*	0.471 (0.231–0.961)	0.038*
>6.03	176	25				

Abbreviations: EBRT, external beam radiation therapy; HDR‐ICBT, high‐dose rate intracavitary brachytherapy.

* *p* < 0.05;

** *p* < 0.01.

### 3.3 Nomograms construction and performance comparisons

**FIGURE 3:**
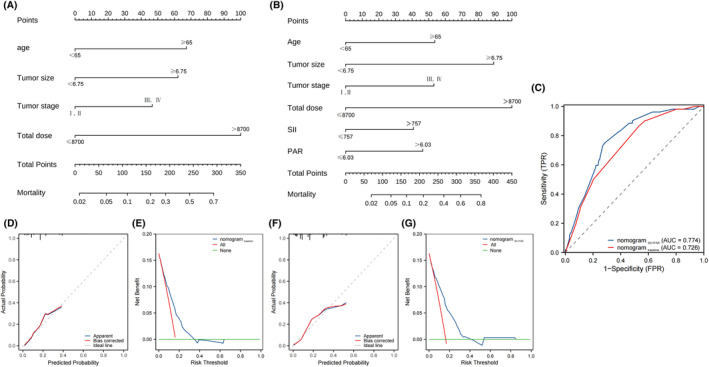
Comparison of two nomograms. (A) baseline nomogram; (B) nomogram with SII and PAR; (C) AUC comparison in two nomograms; (D) The calibration curve of baseline model; (E) DCA curve of baseline mode; (F) The calibration curve of nomogram with SII and PAR; (G) DCA curve of nomogram with SII and PAR.

First, risk factors in univariate analysis (*p* < 0.05) were put into backward stepwise selection to build predictive models. We constructed a baseline nomogram model with age, tumor size, stage, and total radiation dose to predict RC occurrence (Figure 3A). The AUC value of the baseline nomogram was 0.726 (Figure 3C). Then, we added SII and PAR to the above‐mentioned factors to build another predictive model with a higher AUC value of 0.774 (Figure 3B). The nomogram with SII and PAR showed a better prediction ability compared to the baseline nomogram by the Delong test (*p* = 0.02) (Figure 3C). Moreover, the stability of the nomogram with SII and PAR was well validated by Five‐fold validation (Table S1), indicating the critical roles of SII and PAR in RC prediction. The calibration and DCA curves also showed good concordance in both nomograms (Figure 3D–G).

## 4 DISCUSSION

To our knowledge, this was the first study to construct an RC prediction model based on inflammation biomarkers in local advanced CC treated with radiotherapy. We first comprehensively screened out significant clinical variables associated with RC, including age, tumor size, stages, total radiation dose, pelvic radiation dose, SII, PLR, and PAR by univariate analysis. Then, age, tumor size, stage, total radiation dose, and PAR were further selected as independent factors by multivariate analysis. Based on backward stepwise selection, we constructed and compared two prediction models with or without SII and PAR to evaluate the roles of SII and PAR in RC prediction. We demonstrated the model with SII and PAR had higher superiority. Our model could stratify who needs firsthand awareness of preventable measures to avoid RC occurrence before radiotherapy.

PAR and SII are two widely appreciated systemic immune‐inflammation indices due to their integrative characteristics of low cost and easy‐to‐measure.^18^, ^19^ PAR, the PLT divided by the ALB, was proven to be positively closely related to the severity and prognosis of many inflammatory diseases, such as ankylosing spondylitis disease,^20^ acute kidney injury,^21^ periprosthetic joint infection.^22^ On the other hand, SII, calculated from the counts of lymphocytes, neutrophils, and platelets, has also been used to assess the severity of acute pancreatitis and rheumatoid arthritis.^23^, ^24^ However, few studies focused on PAR and SII levels before inflammatory processes. Shan et al. found higher pretreatment SII (*p* = 0.023) and PLR (*p* = 0.001) could predict a higher possibility of radiation‐induced lung injury by univariate analysis.^25^ Surprisingly, our study found higher pretreatment PAR and SII levels were negatively correlated with RC in CC patients. There might be some reasons contributing to it. First, the previous study implied the positive correlation between pretreatment inflammatory biomarkers and radiation‐induced lung injury only based on univariate analysis with small samples of 172 patients. Second, the heterogeneity of different diseases might be another reason. The previous study focused on lung injury of lung cancer patients. Our study targeted adjacent normal tissue to the cervix, which is the bladder. Since literature has limited supporting information, more deep experiments and clinical trials with larger sample sizes are needed to underline the potential mechanism.

DNA damage and patient heterogeneity are critical for understanding RC occurrence.^26^ With the first dose of radiation, there is immediate tissue damage, production of short‐lived free radicals, activation of local inflammation, and change in cellular function, which irreversible breaks in cellular DNA. Massive DNA is damaged in cells as the dose increases through reactive oxygen species (ROS) induction. ROS causes activation of pro‐inflammatory factors and substantial damage to DNA, proteins, lipids, and RNA within the cell, leading to cellular dysfunction and cell death.^27^, ^28^, ^29^, ^30^ DNA repair system plays a crucial role in normal tissue tolerance to radiation.^26^ Patients' genetic conditions contribute to different abilities in DNA repair mechanisms. For example, ataxia telangiectasia is closely related to the mutation of the ataxia‐telangiectasia mutated gene. Patients with the disease are more likely to develop severe complications after radiation because they cannot repair their DNA.^31^ Therefore, patients' genetic diversity is responsible for different sensitivities of normal tissue to radiotherapy, which explains why side effects are not equally the same.

As far as we know, risk factors associated with RC development in CC patients treated with radiotherapy haven't been well investigated in the literature. Our study found that besides PAR and SII, clinical risk factors including age, tumor size, stage, and total radiation dose, also affected RC occurrence. In clinical practice, each variable alone could only play a limited role in assessing the risk of research endpoints. Considering a problem from multiple angles could lead to breakthroughs. Integrating several independent risk factors by the nomogram is an excellent way to improve prediction ability and facilitate clinicians making accurate and effective decisions. This research built two clinical nomogram models. One is the baseline nomogram with age, tumor size, stage, and total dose. The other one included SII, PAR, and the abovementioned factors to improve its prediction ability (AUC_baseline_ = 0.726 AUC_PAR+SII_ = 0.774, *p*<0.05). We added PAR and SII to broaden applicability in the clinic. The calibration and DCA curves showed good discrimination.

PAR and SII, which are economical and practical, could be widely applied in clinical settings. The nomogram with PAR and SII exhibited a significantly more robust capability in RC risk stratification for CC patients treated with radiotherapy, guiding personalized treatment plans and nursing care. However, our study had several limitations. First, it is a retrospective, non‐randomized design study with only Chinese participants. Second, preexisting medical conditions, such as chemo‐drug usage and tumor position, are essential factors in developing RC, which our study still needs to discuss. Third, our study did not distinguish between acute and chronic RCS. Fourth, PAR and SII were assessed by single measurements at admission for the initial diagnosis. Multi‐center and prospective studies are required to confirm our results. Further exploration is also needed to investigate the correlation between inflammatory indicators and the severity of radiation cystitis.

## 5 CONCLUSION

Therefore, PAR and SII, especially PAR, could be valued for CC patients with radiation therapy. The nomogram based on PAR and SII could identify patients who need more extra intervention and nursing care to prevent bladder radiation damage.

## AUTHOR CONTRIBUTIONS

**Jie Lin:** Data curation (equal); methodology (equal); writing – original draft (equal); writing – review and editing (equal). **Jiexiang Lin:** Methodology (equal); writing – review and editing (equal). **Linying Liu:** Formal analysis (equal). **Ning Xie:** Software (equal); writing – review and editing (equal). **Haijuan Yu:** Writing – original draft (equal). **Sufang Deng:** Visualization (equal). **Yang Sun:** Conceptualization (equal).

## FUNDING INFORMATION

This study was supported by the Major Scientific Research Program for Young and Middle‐aged Health Professionals of Fujian Province, China (Grant No. 2022ZQNZD008) and the High‐level Talents Training Project of Fujian Cancer Hospital (Grant No. 2022YNG04).

## CONFLICT OF INTEREST STATEMENT

The authors declare that the research was conducted in the absence of any commercial or financial relationships that could be construed as a potential conflict of interest.

## ETHICS STATEMENT

This is an observational and retrospective study and was approved by the Fujian Cancer Hospital Ethics Committee (K2023‐422‐01).

## CONSENT

Oral informed consent was obtained from all individual participants included in the study.

## Supporting information


Table S1.

## Data Availability

The data analyzed during the current study are available in the Yang Sun repository.
