# Supplementary material for: A novel nomogram based on inflammation biomarkers for predicting radiation cystitis in patients with local advanced cervical cancer
Source: Cancer Med. 2024 May 24;13(10):e7245. doi: 10.1002/cam4.7245 (PMC11117193; doi:10.1002/cam4.7245)
Supplement: Supplementary file 1 — Table S1. [file CAM4-13-e7245-s001.docx]

Table S1 The five-fold validation in nomogram with SII and PAR

| Fold | Cox-Snell R2 | Nagelkerke R2 | accuracy | precision | recall | F measure | AUC |
| --- | --- | --- | --- | --- | --- | --- | --- |
| 1 | 0.41009 | 0.42034 | 0.89062 | 0.50000 | 0.14286 | 0.22223 | 0.7630 |
| 2 | 0.43245 | 0.44724 | 0.75000 | 0.20000 | 0.07692 | 0.11111 | 0.7050 |
| 3 | 0.37264 | 0.38338 | 0.82540 | - | 0.00000 | - | 0.7610 |
| 4 | 0.36955 | 0.38031 | 0.82812 | 0.00000 | 0.00000 | - | 0.8000 |
| 5 | 0.40563 | 0.41808 | 0.81250 | 0.33333 | 0.09091 | 0.14286 | 0.7400 |
| mean | 0.39807 | 0.40987 | 0.82133 | - | 0.06214 | - | 0.7538 |
